# Machine Learning-Assisted Speech Analysis for Early Detection of Parkinson’s Disease: A Study on Speaker Diarization and Classification Techniques

**DOI:** 10.3390/s24051499

**Published:** 2024-02-26

**Authors:** Michele Giuseppe Di Cesare, David Perpetuini, Daniela Cardone, Arcangelo Merla

**Affiliations:** Department of Engineering and Geology, University G. D’Annunzio of Chieti-Pescara, 65127 Pescara, Italy; michelegiuseppe.dicesare@studenti.unich.it (M.G.D.C.); d.cardone@unich.it (D.C.); arcangelo.merla@unich.it (A.M.)

**Keywords:** speech analysis, speaker diarization, machine learning, Parkinson’s disease

## Abstract

Parkinson’s disease (PD) is a neurodegenerative disorder characterized by a range of motor and non-motor symptoms. One of the notable non-motor symptoms of PD is the presence of vocal disorders, attributed to the underlying pathophysiological changes in the neural control of the laryngeal and vocal tract musculature. From this perspective, the integration of machine learning (ML) techniques in the analysis of speech signals has significantly contributed to the detection and diagnosis of PD. Particularly, MEL Frequency Cepstral Coefficients (MFCCs) and Gammatone Frequency Cepstral Coefficients (GTCCs) are both feature extraction techniques commonly used in the field of speech and audio signal processing that could exhibit great potential for vocal disorder identification. This study presents a novel approach to the early detection of PD through ML applied to speech analysis, leveraging both MFCCs and GTCCs. The recordings contained in the Mobile Device Voice Recordings at King’s College London (MDVR-KCL) dataset were used. These recordings were collected from healthy individuals and PD patients while they read a passage and during a spontaneous conversation on the phone. Particularly, the speech data regarding the spontaneous dialogue task were processed through speaker diarization, a technique that partitions an audio stream into homogeneous segments according to speaker identity. The ML applied to MFCCS and GTCCs allowed us to classify PD patients with a test accuracy of 92.3%. This research further demonstrates the potential to employ mobile phones as a non-invasive, cost-effective tool for the early detection of PD, significantly improving patient prognosis and quality of life.

## 1. Introduction

The integration of edge computing into smart healthcare systems represents a significant advancement in modern healthcare, promising to revolutionize the industry by bringing computation closer to the source of data generation. Its deployment for smart healthcare has already been assessed, highlighting the security risks encountered and proposing solutions to address said risks, thus providing a credible framework for smart healthcare [1]. Furthermore, with the integration of artificial intelligence, edge computing has developed into the concept of edge intelligence, paving the way for highly compelling applications such as real-time critical healthcare systems [2]. Notably, the integration of edge computing and smartphones into these structures has revealed new frontiers for efficient data collection, the early detection of health issues, and the delivery of healthcare services. In fact, smartphones, with their widespread adoption and array of sensors, have emerged as indispensable tools for aiding in the diagnosis, treatment, and continuous monitoring of individuals’ health. They can capture data on various physiological parameters and easily connect to wearable devices and sensors, creating a seamless ecosystem for continuous data collection. Importantly, they have found wide application in the field of voice analysis for the early detection of health issues. This is due to their optimal microphones, which have been evaluated for their fidelity and accuracy in the acoustic measurement of voices [3]. Particularly, the suitability of smartphone microphones for capturing voice recordings, even in the presence of ambient noise, and their capability to successfully record lung sounds, making auscultation feasible, are widely demonstrated in the literature [4,5]. Moreover, they have been found to be adequate for the digitization of pathologic voices, making them valuable for clinical voice research [6]. The high performance of smartphone microphones has been further proven in studies showing how they outperform standard external microphones in recording and analyzing normal and dysphonic voices [7]. Lastly, it was demonstrated that smartphones are useful in restoring pathological voices and allowing greater access to voice therapy for patients with dysphonia [8]. It should be noted, though, that challenges such as device limitations in continuous voice tracking have been identified, highlighting the need for further technological advancements to overcome these barriers [9]. From this perspective, the development and the investigation of the effectiveness of diarization algorithms to identify different speakers during a conversation are crucial in order to also properly assess vocal heath status in ecological situations, such as phone calls.

Importantly, vocal analysis often integrates machine learning (ML) techniques with the aim to improve the classification performance between healthy and pathological voices [10]. The effectiveness of ML techniques combined with vocal analysis has been explored for the classification of health conditions in several pathologies, such as Parkinson’s disease (PD) [11].

Specifically, PD is a neurodegenerative disorder characterized by a range of motor and non-motor symptoms, including tremors, bradykinesia, rigidity, and postural instability [12,13]. One of the notable non-motor symptoms of PD is the presence of vocal disorders, which significantly impact speech and communication abilities in affected individuals. The vocal disorders associated with PD are collectively termed hypokinetic dysarthria and are attributed to the underlying pathophysiological changes in the neural control of the laryngeal and vocal tract musculature [14]. Additionally, the dysarthria and vocal tremor observed in PD are linked to modifications in speech and voices that resemble those seen in the normal aging process, albeit with specific differences in prosody and habitual frequency, which have a significant negative impact on the quality of life of individuals with PD [15]. At the time of this study, the diagnostic protocol utilized for PD assessment relied upon the MDS-Unified Parkinson’s Disease Rating Scale (MDS-UPDRS). This scale, administered by physicians, encompasses a structured questionnaire aimed at capturing various facets of patients’ daily experiences alongside a comprehensive motor examination. Notably, within the motor examination part, clinicians are tasked with evaluating tremor severity solely through visual inspection. This presents a notable limitation, as the severity of tremors is known to be associated with variations in tremor amplitude, a quantitative metric often measured in centimeters. Consequently, this reliance on visual assessment renders the current evaluation method inherently approximate. Given the previous considerations, the purpose of this study is to propose an ML-based analysis of voice recording acquired by means of a smartphone and preprocessed with a speaker diarization algorithm, able to classify healthy control (HC) and PD patients. Particularly, the diarization algorithm allowed us to split the audio recording into distinct speaker-specific parts, thus identifying the PD patients during a phone conversation. The ML frameworks were fed using the MEL Frequency Cepstral Coefficients (MFCC) and Gammatone Frequency Cepstral Coefficients (GTCCs), which are both feature extraction techniques commonly used in the field of speech and audio signal processing [16,17]. In fact, both MFCCs and GTCCs are widely used in various audio processing applications, and their effectiveness in capturing the essential characteristics of audio signals has been demonstrated in tasks such as speaker identification, emotion recognition, environmental sound classification, speech recognition, and voice disorder assessment [18]. It is imperative to acknowledge that, despite the promise of using vocal analysis as a means of assessing PD, caution must be exercised during data acquisition, since the presence of noise in the recordings poses a significant obstacle to the classifier’s accuracy, and furthermore, the quantity of data collected is of paramount importance, as an adequate volume of data is necessary to facilitate unbiased classification. 

In conclusion, the novelty of the proposed approach relies in the combination of MFCCs and GTCCs, which has been poorly investigated so far, and on the employment of the diarization algorithm to identify speakers during a phone call, in order to also foster the application of voice analysis for vocal disorder assessment for ecological applications.

## 2. Materials and Methods

This study was conducted using the freely available Mobile Device Voice Recordings at King’s College London (MDVR-KCL) dataset [19], which comprises a total of 37 voice recordings, 21 from HC and 16 from PD patients. As their first task, subjects were asked to make a call and read one of the following paragraphs:

(1) “The North Wind and the Sun were disputing, which was the stronger, when a traveler came along wrapped in a warm cloak. They agreed that the one who first succeeded in making the traveler take his cloak off should be considered stronger than the other. Then the North Wind blew as hard as he could, but the more he blew the more closely did the traveler fold his cloak around him; and at last, the North Wind gave up the attempt. Then the Sun shone out warmly, and immediately the traveler took off his cloak. And so, the North Wind was obliged to confess that the Sun was the stronger of the two”.
(2) “This is because there is less scattering of blue light as the atmospheric path length and consequently the degree of scattering of the incoming radiation is reduced. For the same reason, the sun appears to be whiter and less orange-coloured as the observer’s altitude increases; this is because a greater proportion of the sunlight comes directly to the observer’s eye. Figure 5.7 is a schematic representation of the path of electromagnetic energy in the visible spectrum as it travels from the sun to the Earth and back again towards a sensor mounted on an orbiting satellite. The paths of waves representing energy prone to scattering (that is, the shorter wavelengths) as it travels from sun to Earth are shown. To the sensor it appears that all the energy has been reflected from point P on the ground whereas, in fact, it has not, because some has been scattered within the atmosphere and has never reached the ground at all”.

As the second task, subjects were asked to have a spontaneous dialogue with the test executor on the other end of the line. 

Both tasks were recorded through the microphone of a Motorola Moto G4 smartphone (launched in 2016). It should be noted that recordings were performed with a ‘Toggle Recording App’ function, which used the same functionalities as the voice recording module used in the i-PROGNOSIS app (https://cordis.europa.eu/project/id/690494, accessed on 5 September 2023). In summary, at the call’s beginning, the Toggle app initiated recordings that ceased when the call concluded. This kind of acquisition allowed recordings to be taken directly through the smartphone microphones, and not through the GSM (Global System for Mobile communications) compressed stream, meaning every audio track had the highest resolution possible, with a sampling rate of 44.1 KHz and a 16-Bit depth. The MDVR-KCL has been deemed the best option for the presented study given the nature of the acquisitions, which ensured the maximum possible fidelity to the real patients’ voices and the presence of recordings from daily tasks such as reading and phone conversations. Particularly, this last feature played a fundamental role in the selection, given its congruence with real-life activities.

Two parallel analyses were conducted to explore the impact of different sampling strategies. Given the limited number of subjects and the high sampling frequency, the first analysis involved sampling the original recordings with no overlap, ensuring that biases were minimized during subsequent classification. This sampling strategy applied only to the reading task recordings, resulting in 3156 and 2044 audio samples for HC and PD patients, respectively. Simple partitioning, however, was not a viable option for the recordings of the spontaneous conversation task, given the presence of a second voice in the audio files; thus, a more complex approach was required. In this case, the implementation of a speaker diarization algorithm preceded the ML classification. Speaker diarization is a method for segmenting audio streams into distinct speaker-specific intervals. The algorithm involves the use of k-means clustering in conjunction with an x-vector pretrained model. X-vectors, obtained from deep neural networks processing MFCCs, encapsulate unique speaker characteristics. K-means clustering is then applied, iteratively refining centroids to group x-vectors into distinct clusters, effectively associating each segment with a specific speaker. In the diarization process, the audio stream is initially divided into shorter intervals, from which x-vectors are extracted and subsequently clustered and processed. Post-processing techniques, such as smoothing, handle challenges like overlapping segments, ensuring a coherent and accurate diarization output. It is important to note that, when applying this technique, the number of clusters to identify should always be set to the number of speakers plus one. This is carried out in order to store any background noise in a unique, separate cluster, thereby enhancing the accuracy of the process and the quality of the output audio streams. However, in the current case, recordings were made directly on the call, resulting in no background noise. Thus, the algorithm was simplified, necessitating the inclusion of a number of clusters equal to the number of speakers. Furthermore, the complete absence of noise enhanced the performance of the algorithm, with the process resulting in successful isolation, with 100% accuracy, of the voices of the subjects from the voice of the supervisors on the other end of the call, storing them into distinct audio files without altering their features or content. This led to 273 voice audio samples for HC subjects and 247 voice audio samples for individuals with PD. Lastly, it is paramount to highlight that, without the speaker diarization process, the classification in the spontaneous conversations would have been rendered untruthful, since the presence of the second healthy speakers in the conversations with PD patients would have certainly represented bias in the classification, altering the results obtained.

The ML models tested in this study were fed using MFCCs and GTCCs. With numerous coefficients in each, MFCCs provide a thorough depiction of the spectral envelope, addressing redundancies and offering nuanced insights into vocal characteristics. Stemming from the Gammatone filterbank, GTCCs offer a physiologically accurate representation of the auditory system’s frequency response. 

MFCC is a method of representing the power spectrum of a sound over a short period of time, using the human auditory system’s reaction to sound as a basis. The process involves first applying Fourier transform to a signal, followed by calculating the logarithm of the magnitude of the resulting Fourier transform. Next, there is a conversion to the MEL scale, which is a perceptual scale where listeners perceive pitches to be equally spaced from each other. Ultimately, the MEL log spectrum undergoes discrete cosine conversion in order to obtain the MFCCs [16].

Conversely, GTCC is a technique for extracting features that is influenced by the way the human auditory system responds to sound. The gammatone filterbank is designed to mimic the frequency response of the basilar membrane found in the human ear. The gammatone filterbank is first applied to the signal, followed by obtaining the logarithm of the magnitude of the filterbank outputs. Finally, discrete cosine transform is used to produce the cepstral coefficients [17,20]. 

Both feature sets contribute to a well-balanced representation of crucial spectral details without unnecessary complexity. Extracted from audio signals, these features aim to capture significant variations in the voice, making them suitable for tasks such as identifying speech impairments, recognizing emotions, and managing conditions like asthma. In the healthcare domain, both MFCCs and GTCCs find application in diverse areas, including the detection of speech impairments, emotion recognition, and the management of asthma [21,22,23]. The analyses described were performed with 13 coefficients each, extracted and normalized with a z-score algorithm, after the application of a pre-emphasis filter on the recording, aligning with current common practice [24]. 

**Figure 1:**
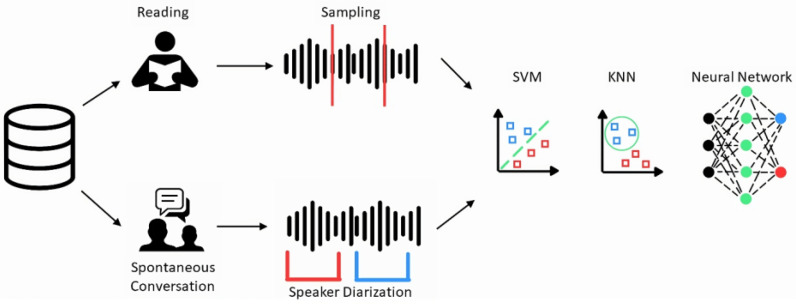
Processing pipeline of the analyses performed in the study. Recordings from both tasks were classified through all three ML models represented. Concerning the representation of the ML algorithms, the blue and red squares in the SVM and KNN representation are indicative of the two different groups. Regarding the Neural Network, the black circles are the first layer of the machinery, the green circles represent the inner layers, whereas the red and blue circles are indicative of the two groups provided as output by the model.

Both MFCCs and GTCCs have been employed separately in the assessment of PD patients from voice recordings [25,26]. Furthermore, studies have shown how cepstral coefficients can be defined as new quantitative biomarkers for the assessment of this specific disease, and how GTCCs outperform every other audio feature in the field of speech analysis [27,28]. Hence, employing both MFCCs and GTCCs could potentially offer better performance in identifying PD than using either set of coefficients alone. It should also be noted that even considering both sets of coefficients, the number of samples is much larger than the number of predictors; hence, this choice does not negatively affect the classifications. Once the coefficients for each recording were extracted, separate tables, designed for utilization in ML applications, were prepared: one specifically for training, and the other for testing purposes. It is crucial to note that to avoid any bias in the resulting accuracies, every table was built selecting the same number of random samples for both the HC and the PD classes, thus conferring evenness. Lastly, classifications were performed through Support Vector Machine (SVM), k-nearest neighbor (KNN), and neural network models, which are widely utilized in the field of speech analysis [29,30]. Specifically, the SMV model adopted a ‘One-vs-one’ strategy for multiclass classification [31], while the KNN model was set to have one neighbor and Euclidean distance as classification metrics. Lastly, the neural network chosen was the wide model configured with an intermediate layer size of 100. The training was performed using nested cross-validation (nCV), where the dataset is divided into folds, and the model is trained repeatedly on all but one fold of the data. The inner loop determines the most effective hyperparameters via validation, while the outer loop assesses the model’s performance over iterations through testing [32,33,34]. Specifically, 5-fold cross validation was implemented. The complete processing pipeline is represented in Figure 1.

Analyses were performed through MATLAB 2023b©. Notably, the number of predictors was significantly lower than the number of samples, hence allowing us to reduce the possibility of an overfitting effect.

Furthermore, a *t*-test was performed for both MFCCs and GTCCs extracted from the diarized recording to examine the differences between PD and HC. This analysis aims at gaining a more comprehensive understanding of how differences in the cepstral coefficients are associated with the health status of the individual.

## 3. Results

### 3.1. Reading Task—Standard Sampling

For the reading task, given that the only voices present in the recordings were those of the subjects, partitioning the data was the only viable option to increase the dataset size. Following a random selection process, a training set comprising 3000 samples and a testing set of 300 samples were used for processing with various SVM and KNN models. As mentioned in the previous sections, tables were evenly constructed, with both containing 50% samples from HC and 50% samples from PD patients. The results from the various ML models are presented in detail in Table 1.

**Figure 2:**
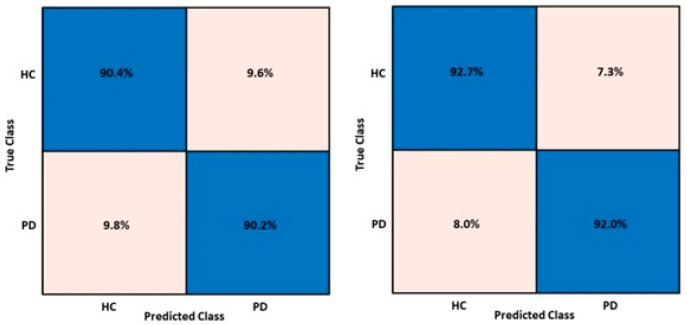
Training and test confusion matrices for the wide neural network model implemented with MFCCs and GTCCs.

Of the multiple trials, the confusion matrices reporting the best results for the best performing model for the training and test performances are shown in Figure 2.

### 3.2. Spontaneous Conversation Task—Speaker Diarization

For the spontaneous conversation task, after the diarization process and random selection, a training set comprising 400 samples and a testing set of 40 samples were utilized for processing with different SVM and KNN models. As mentioned in the previous sections, tables were evenly constructed, with both containing 50% samples from HC and 50% samples from PD patients. The results from the diverse ML models are reported in detail in Table 2.

**Figure 3:**
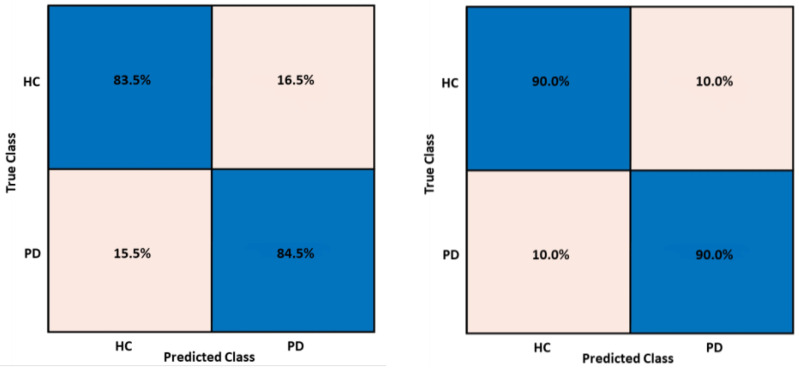
Training and test confusion matrices for the Cubic SVM model implemented with MFCCs and GTCCs.

Of the multiple trials, the confusion matrices reporting the best results for the best performing model for training and test performances are shown in Figure 3. 

**Figure 4:**
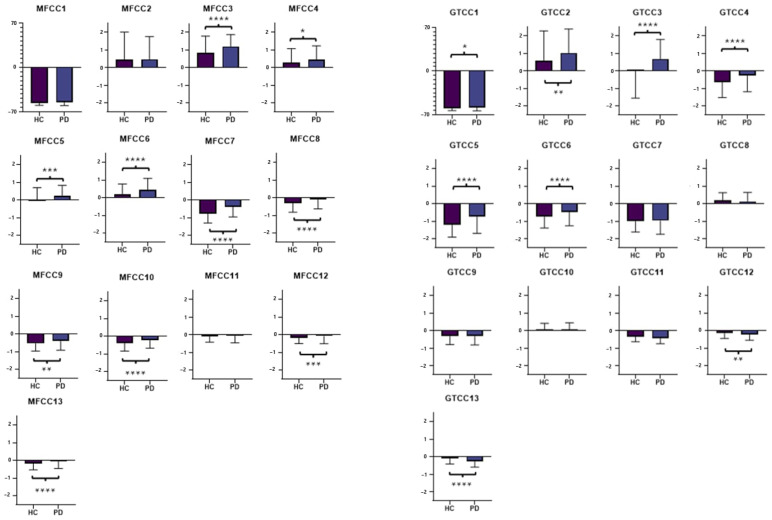
Cumulative distributions of MFCCs and GTCCs extracted from the diarized samples and indications about the results of the unpaired *t*-test performed: * *p* < 0.05; ** *p* < 0.01; *** *p* < 0.001; **** *p* < 0.0001.

The results of the unpaired *t*-test revealed significant differences in the distribution of most of the cepstral coefficients, as reported in Figure 4. Particularly, while MFCCs had the most groups with significant differences, the best results in the classifications were obtained by leveraging GTCCs, meaning that their differences held higher weight in ML processing. 

## 4. Discussion

This study employed a multifaceted approach to discern between HC and PD patients, relying solely on vocal samples processed through a simple partition or a speaker diarization algorithm.

The results show that, while all ML models considered can be valuable in the field of speech analysis, SVM and neural networks prove to be more effective than KNN in the described task. Furthermore, although all classification accuracies are comparable, utilizing a combination of MFCCs and GTCCs proves to be the optimal choice, yielding better results at a comparable computational cost. For instance, in the case of SVM for the second task, a total cost of 66 was registered when utilizing only GTCCs as features, achieving a test accuracy of 82.5%, compared to a cost of 68 assessed when classifying through the same model but leveraging both MFCCs and GTCCs, reporting a test accuracy of 90.0%. The inferential analysis supports the optimal results obtained through ML classifications, highlighting significant differences almost in the totality of the *t*-test performed. 

While the results observed in the reading task classification are generally higher than the ones obtained for the spontaneous dialogue, it is important to note the different number of samples in the tables involved in the process. Previous studies demonstrated the possibility of classifying PD patients from HCs by employing the cepstral coefficient. For instance, Benba et al. employed MFCC and Support Vector Machine (SVM) to analyze voiceprints for detecting patients with PD, demonstrating the effectiveness of MFCC in differentiating individuals with PD from healthy subjects, reaching an accuracy of 91.7% [18]. Moreover, Boualoulou et al. proposed a classification on a small dataset of 38 vocal recordings—20 from PD patients and 18 from healthy controls—first applying an algorithm of empirical mode decomposition on the signals, and then, leveraging MFCCs and a KNN model, reaching an accuracy of 86.7% [35]. Later on, in another study, the same technique was applied in combination with discrete wavelet transform to two different datasets, and both MFCCs and GTCCs were extracted. Classifications were conducted with more advanced methods such as convolutional neural networks and long short-term memory networks, achieving accuracies of 96.55% and 100% on the different datasets, employing in both cases only the GTCCs [26]. However, in none of these cases were MFCCs and GTCCs employed together, and none of the datasets analyzed contained conversations between subjects or recordings acquired during a phone call. It should be highlighted that the novelty of the present study relies on the possibility of identifying PD using vocal recordings performed by smartphones, whereas the previous studies used professional recordings. Furthermore, employing speaker diarization algorithms unlocks the potential for real-life applications, not having limits to the number of speakers it manages to identify, thus making it appliable to daily conversations. Another highlight lies in the nature of the tasks performed, both simulating common situations, which are easily reproducible, and thus presenting the possibility of building new datasets which could be worked on with the goal of improving research and results in the field of vocal analysis. 

The first limitation of the conducted research, however, lies in the limited number of subjects. A larger number of patients participating in the data acquisitions would have influenced the study differently, removing the need for partition in the first task, thus achieving more realistic results. Plus, assuming that the slightly lower accuracies registered in the spontaneous conversation classifications are once again attributed to the number of samples, increasing the number of subjects would likely also enhance the results obtained. However, it must be noted that performing the same study on a larger and more diverse dataset would have probably lowered the accuracies obtained in the absence of a proper training phase. This places a question mark on the nature of the training required for the construction of a model with high performance on a larger scale. Another point to highlight regarding the specific dataset employed is related to the lack of the selection criteria for the passages read by participants. For instance, the linguistic complexity of the passages (e.g., vocabulary difficulty, sentence structure) can influence the cognitive load on participants, potentially affecting their speech production. Passages with varying complexity levels can elicit speech that captures different aspects of PD-related speech impairments. Moreover, a selection based on phonetic content could aim to ensure that the passages cover a broad range of phonemes and phonetic contexts. This comprehensive coverage can help in analyzing how PD affects different aspects of speech production. Regarding the emotional and semantic content of the passages, the different emotions evoked could require deep semantic processing that might elicit differences in prosody and articulation. Notably, the length of the passages and their readability can affect participant fatigue and engagement, potentially influencing their speech characteristics. An optimal length would ensure that participants do not become fatigued, which could affect speech production. Finally, familiarity with the passage content can affect a participant’s comfort level and speech naturalness. Passages that are relevant and interesting to participants might elicit more natural speech patterns; hence, choosing passages that are too familiar or too relevant to the participants’ personal experiences might lead to variations in expressiveness that are not directly related to PD symptoms. From this perspective, the passages should be selected based on a comprehensive understanding of PD’s impact on speech, considering the balance between uniformity (to ensure comparability across participants) and diversity (to cover a broad range of speech characteristics). Further studies should investigate how the passages’ selection could impact the classification outcomes.

Potential sources of errors in the classification accuracy might be represented by subjects with minor symptoms affecting their vocal apparatus. Although this case cannot be explored in the presented study, given the absence of such information in the analyzed dataset, this possibility should be considered in future studies, with the goal of developing a stronger classifier able to take into account the severity of vocal disorders and to identify the progression of the disease.

Furthermore, high recording quality with no background noise may not be guaranteed in other applications or datasets, emphasizing the necessity for more advanced speaker diarization algorithms, which should employ a separate cluster for storing said noise, or even a different, more solid model than k-means clustering. This is especially true in real-life scenarios when not only is a high noise component present, but there is also a variable number of speakers in a single conversation. Moreover, considering real-life applications, the classifiers implemented should be open to the possibility of the presence of more than one kind of disease, meaning they should be capable of accurately assessing various kinds of shadows in vocal distortions. This is particularly important, as different pathologies may exhibit similar alterations in how the voice is perceived, potentially weakening the effectiveness of cepstral coefficients as features utilized by ML models. A potential solution to address this challenge lies in enhancing the strength of the coefficients. Utilizing a higher number of coefficients, in fact, would result in a more accurate representation of vocal characteristics, thereby improving the overall robustness and efficacy of the classifier. However, it should be noted that such an application would once again require a proper training phase, which means a comprehensive dataset of these different kinds of diseases should be used. Furthermore, regarding possible questions about the quality of recordings performed through mobile devices, it should be highlighted that smartphone microphones have been deemed to perform exceptionally well when the goals are healthcare applications [36,37]. 

Still regarding limitations, it is important to understand that classification performed is just a simple assessment of the disease, and thus, is not comparable with the evaluation carried out by the physician with the support of the MDS-UPDRS. However, this study is meant to be a first step towards automated classification of the severity of the disease, and the final goal should be the complete replacement of the scale, establishing a new model for the disease’s evaluation.

Future developments should be aimed at overcoming the reported limitations, employing solutions such as improved diarization algorithms, based on models more advanced than k-means for the clustering of different voices. Furthermore, a deep learning approach should be conducted, aimed at constructing a classification model which performs well without the need for training. This approach represents an initial stride towards the real-time assessment of an individual’s health through straightforward vocal recordings. These recordings could play a dual role by not only serving to refine and enhance the model, but also contributing to the creation of an ultimate system. Such a system would possess the capability not only to offer immediate diagnoses but also to detect subtle changes that might indicate the potential development of a specific disease. In order to enhance the model’s performance, further predictors could be considered. Specifically, the adoption of more than 13 cepstral coefficients as classification features for a widened representation of the vocal characteristics could be implemented. Moreover, integrating additional features or data types beyond MFCCs and GTCCs can significantly enhance a model’s predictive power for identifying vocal disorders associated with PD. These features can provide complementary information about speech production, articulation, and other characteristics affected by PD. For instance, prosodic features such as pitch variation, speech rate, pause duration, and intonation patterns can be used. In addition, voice quality features reflecting the characteristics of the voice signal itself, including jitter (frequency variation), shimmer (amplitude variation), harmonics-to-noise ratio (HNR), and voice breaks could be used as predictors. Furthermore, articulatory features which provide insights into the movements and positions of the articulators (e.g., tongue, jaw, lips) during speech production could be integrated into the model. Moreover, non-linear dynamic features (e.g., detrended fluctuation analysis, recurrence quantification analysis, and Lyapunov exponents) that can capture the complexity and variability of speech signals, revealing subtle changes in speech production mechanisms, can be exploited to increase the performance of the model. Importantly, it should be highlighted that incorporating these additional features and data types requires enlarging the dataset in order to avoid overfitting effects related to the elevated number of predictors with respect to the classes’ numerosity.

In fact, another crucial development would be the construction of a larger dataset of vocal recordings, for the specific purpose of classifying not only the PD, but also other similar neurodegenerative diseases. This would obviously be constricted by ethical considerations and privacy concerns [38], which require careful consideration and can only be addressed by ensuring transparency, data security, and user privacy preferences at the moment of the acquisitions. 

Moreover, in the near future, serious consideration should be given to implementing a portable app. Such an application could analyze diverse recordings captured by the individual and, after undergoing a sufficient number of processes, offer a straightforward health evaluation. This concept closely aligns with the heart-monitoring technologies found in smartwatches, offering simple indications of one’s health. This approach brings a better health status understanding directly to the individual, eliminating the need for direct consultation with a physician, thus enabling a simple at-home assessment. Furthermore, it is not difficult to imagine a more complex version of the app to be used in a clinical environment in difficult cases as an aiding tool for physicians which could also keep track of examined cases, also working as a reference for complex diagnosis, without ignoring the privacy concerns previously cited. From this perspective, it should be noted that using mobile phones for collecting sensitive health-related data raises significant ethical considerations and privacy concerns. The convenience and ubiquity of mobile phones make them powerful tools for health monitoring and data collection, but they also pose risks related to confidentiality, data security, and user consent. Firstly, individuals must fully understand what data are being collected, how they will be used, and who will have access to them. To address this concern, it is necessary to implement clear and comprehensible consent processes, possibly through the app interface, where users can review and agree to the terms before participating. Ensuring that consent is freely given and can be withdrawn at any time is crucial. Moreover, employing strong encryption for data transmission and storage is necessary, as are adhering to best practices for cybersecurity and ensuring that data are anonymized or de-identified to protect participant identity. Regular security audits and compliance with standards like HIPAA (Health Insurance Portability and Accountability Act) in the U.S., GDPR (General Data Protection Regulation) in the EU, and other relevant data protection regulations are essential.

However, more immediate steps should be taken in the direction of clinical trials. Since voice analysis has found wide applications only in recent years, there is a lack of employment of said instruments in real-time clinical applications. This poses an important obstacle to the advancement of studies in the field and should be the first limitation to address. Moreover, given the severity of the disease in question, it is easy to imagine that complementary at-home observations of patients would enhance the findings of the principal clinical trials. 

In conclusion, these findings show the feasibility of the assessment of PD by leveraging cepstral coefficients and ML models, both already established as powerful tools in the realm of speech analysis. The results obtained show promise in paving the way for a greater degree of understanding of the changes in vocal features in PD patients and in the aided diagnosis of said disease. The strength of the presented study lies in its reproducibility and its utilization of features solely dependent on the voice of the subject. Its effectiveness in addressing minor diseases has already been established [39], thus supporting the potential applicability of the same approach to other neurodegenerative diseases and, more broadly, to any condition where the patient’s voice plays a significant role. This underscores the great potential of the presented approach in speech analysis.

## 5. Conclusions

In conclusion, this study proposed an optimal method for the classification of PD patients and HCs by leveraging vocal recordings acquired while reading or having a spontaneous conversation on the phone. Utilizing diverse ML methods, leveraging novel techniques such as speaker diarization, and working on widely used vocal features such as MFCCs and GTCCs, this research showcases the capacity of this method to glean valuable insights into an individual’s health status from a basic voice recording. This study’s significance is underscored by its effective use of a streamlined set of features, emphasizing the inherent potential in leveraging dated recording devices. By highlighting the untapped reservoir of valuable data accessible through older technologies, this work emphasizes the transition from traditional diagnoses to augmented diagnostic approaches, advocating for the utilization of existing resources to enhance medical assessments.

## Figures and Tables

**Table 1:** Details of performance in the classification of samples from the reading task.

Feature Set	ML Model	Training Accuracy	Test Accuracy	Sensitivity	Specificity	F1-Score
MFCCs	Cubic SVM	86.4%	86.3%	0.86	0.86	0.86
	Fine KNN	89.4%	90.3%	0.90	0.91	0.90
	Wide neural network	88.8%	90.7%	0.93	0.87	0.90
GTCCs	Cubic SVM	87.0%	86.7%	0.86	0.88	0.87
	Fine KNN	87.9%	87.7%	0.88	0.87	0.88
	Wide neural network	87.5%	90.3%	0.93	0.88	0.90
MFCCs + GTCCs	Cubic SVM	90.0%	88.7%	0.90	0.88	0.88
	Fine KNN	89.8%	92.3%	0.93	0.91	0.92
	Wide neural network	90.3%	92.3%	0.92	0.93	0.92

**Table 2:** Details of performance in the classification of samples from the spontaneous conversation task.

Feature Set	ML Model	Training Accuracy	Test Accuracy	Sensitivity	Specificity	F1-Score
MFCCs	Cubic SVM	81.5%	85.0%	0.85	0.85	0.85
	Fine KNN	80.2%	85.0%	0.85	0.85	0.85
	Wide neural network	80.5%	87.5%	0.89	0.86	0.87
GTCCs	Cubic SVM	85.2%	82.5%	0.78	0.88	0.84
	Fine KNN	83.2%	77.5%	0.76	0.79	0.78
	Wide neural network	82.8%	82.5%	0.81	0.84	0.83
MFCCs + GTCCs	Cubic SVM	84.0%	90.0%	0.90	0.90	0.90
	Fine KNN	84.8%	75.0%	0.75	0.75	0.75
	Wide neural network	86.8%	87.5%	0.86	0.89	0.88

## Data Availability

Publicly available datasets were analyzed in this study. This data can be found here: https://zenodo.org/records/2867216 (accessed on 5 September 2023).
